# Notes and Notices

**Published:** 1884-10

**Authors:** 


### NOTES AND NOTICES.

REMOVAL.—The office of this journal has been changed to 129 South Thirteenth Street.

ERRATA.—In Dr. Wells' articles the following errata occur: Page 109, 20th line, for *part* read *fact*; page 235, 22d line, for *part* read *fact*.

MEDICAL HISTORY OF THE SURVIVORS OF THE GREELY EXPEDITION is very interestingly told by Surgeon E. H. Green, in *Medical News*, of September 6th, 1884.

GRANT & WARD OUTDONE.—If some of our backward subscribers do not promptly mail us the funds *due us* there will ere long be a financial cyclone in this office!

ANN ARBOR.—Professor A. C. Cowperthwaite, of Iowa, has been elected to the chair of therapeutics in the Homœopathic Department at Ann Arbor, in place of Dr. H. C. Allen, resigned. A good selection.

AN ANNUAL OCCURRENCE.—"The meeting (of the Institute) just closed was in its social aspect a marked success, but was sadly wanting, as we have intimated, in practical results."—*T. M. S. in N. Y. Med. Times.*

A GENIUS TO FAIL.—Speaking of the causes of failure in life, Tourgee says: "Trying to carry too big a load. I don't know about a professional man's failing if he works, keeps sober, and sleeps at home. Lawyers, ministers, and doctors live on the sins of the people, and, of course, grow fat under reasonable exertion, unless competition is too great. It requires real genius to fail in either of these walks of life."

A NUTRITIOUS AND CONCENTRATED FOOD.—Prof. John Attfield (of London) writes: "Beef peptonoids is by far the most nutritious and concentrated food I have ever met with. Indeed, a palatable and assimilable and in every way acceptable article of food, containing nearly seventy per cent. of purely nutritive nitrogenous material, has never before, to my knowledge, been offered to the medical profession or to the public."

CHICAGO, July 2d, '84.

DEAR DOCTOR:—I have just finished reading the July H. P., and though it is midnight, I yield to a desire to thank you, and through you every contributor to the number. It is a mine of practical gems, every one of which is of the purest water. Who can read them without rejoicing that Hahnemann lived!

Fraternally,

E. A. BALLARD.

A SENSIBLE SUGGESTION.—Materia medica and therapeutics being the special objects of our Institute—although they are two subjects but lightly touched on at our present gatherings—we would have the bureaux of materia medica and clinical medicine report each year, making an equal division of the remaining bureaux to report each alternate year. By clinical medicine we mean all that the words imply, and not a report simply on the practice of medicine. There is no reason why the bureaux of anatomy, pharmæcology, microscopy, etc., need to report every year on account of any marked advancement during that year in these particular branches. We would limit the number of papers and the time so that ample opportunity might be given for discussions. We think a plan modeled somewhat on the above principle would bring out practical results.—*M. S. in N. Y. Med. Times.*

